# The NLRP12 Osteoimmune Checkpoint: Coordinating Inflammatory Signaling and Bone Remodeling

**DOI:** 10.3390/biomedicines14081716

**Published:** 2026-07-30

**Authors:** Vincent G. Yuan

**Affiliations:** The Department of Otolaryngology-Head & Neck Surgery, University of Pittsburgh Medical Center (UPMC), Pittsburgh, PA 15213, USA; vincentyuan@pitt.edu

**Keywords:** NLRP12, osteoimmunology, bone remodeling, osteoclastogenesis, innate immunity, inflammatory bone diseases, osteoimmune checkpoint, precision medicine

## Abstract

Bone remodeling is increasingly recognized as an immunologically regulated process in which inflammatory signaling governs the balance between bone formation and resorption. While extensive efforts have focused on pathways that promote osteoclastogenesis, endogenous mechanisms that restrain inflammatory bone destruction remain less well defined. Here, we propose NLRP12 as a previously underappreciated osteoimmune checkpoint that integrates innate immune regulation with skeletal homeostasis. Emerging evidence demonstrates that NLRP12 suppresses NF-κB and MAPK signaling, antagonizes NLRP3 inflammasome activation, and limits the production of osteoclastogenic cytokines, thereby constraining pathological bone resorption. Beyond its direct effects on osteoclast precursors, NLRP12 may shape the broader bone marrow immune niche through regulation of macrophages, dendritic cells, neutrophils, immunometabolic pathways, and host–microbiota interactions. We synthesize current knowledge linking NLRP12 to osteoclast differentiation, inflammatory bone diseases, and osteoimmune communication, and highlight key unanswered questions regarding its functions in osteoblasts, osteocytes, and skeletal aging. By framing NLRP12 as an important regulator of inflammatory tone within the skeletal microenvironment, we introduce an osteoimmune checkpoint paradigm that provides new conceptual insights into the pathogenesis of osteolytic disorders and identifies opportunities for therapeutic intervention.

## 1. Introduction

Bone is a dynamic organ that undergoes continuous remodeling throughout life to maintain skeletal integrity and mineral homeostasis [1]. This process depends on the coordinated activities of bone-resorbing osteoclasts and bone-forming osteoblasts, which together preserve a delicate balance between bone destruction and regeneration [1,2]. The emergence of osteoimmunology has transformed our understanding of skeletal biology by revealing extensive bidirectional communication between immune cells and bone-resident cells [3]. Cytokines, chemokines, and innate immune signaling pathways influence osteoclast differentiation and function, while skeletal cells actively participate in immune regulation within the bone marrow microenvironment [4,5].

Under physiological conditions, inflammatory signaling contributes to normal bone turnover and tissue repair [6]. However, persistent or excessive immune activation disrupts bone homeostasis by promoting osteoclastogenesis and pathological bone resorption [7]. Pro-inflammatory mediators amplify osteoclastogenic signaling cascades, accelerating bone resorption [8,9]. While considerable attention has focused on pathways that initiate inflammatory bone destruction, considerably less is known about endogenous mechanisms that restrain these responses and preserve skeletal integrity [10]. Analogous to immune checkpoint pathways that maintain immune homeostasis, the concept of an osteoimmune checkpoint has recently emerged as a useful framework for understanding intrinsic regulatory mechanisms that limit inflammation-induced bone loss. Osteoclast differentiation is governed by a conserved NF-κB–MAPK signaling network that integrates receptor activator of nuclear factor-κB ligand (RANKL), tumor necrosis factor-α (TNF-α), and interleukin-1β (IL-1β) signaling to activate c-Fos- and nuclear factor of activated T cells 1 (NFATc1)-dependent transcriptional programs [11,12].

Among the intracellular regulators of innate immunity, nucleotide-binding oligomerization domain (NOD)-like receptors (NLRs) have emerged as critical modulators of inflammatory responses [13]. Although several members of the NLR family, particularly NLRP3 [14,15,16], have been extensively investigated as drivers of inflammatory diseases, NLRP12 has received comparatively limited attention [17,18]. Originally identified as Monarch-1 and subsequently classified as NLRP12, this receptor contains an N-terminal pyrin domain, a central nucleotide-binding NACHT domain, and C-terminal leucine-rich repeats [19,20]. NLRP12 is predominantly expressed in myeloid cells, including monocytes, macrophages, dendritic cells, and neutrophils, where it regulates multiple innate immune signaling pathways [21,22,23,24]. Unlike several other pattern-recognition receptors, however, no definitive endogenous or microbial ligand has yet been identified for NLRP12, suggesting that it primarily functions as a context-dependent regulator of inflammatory signaling rather than as a classical ligand-specific receptor [19,25].

Accumulating evidence indicates that NLRP12 serves as a broad modulator of inflammatory signaling by restraining canonical and non-canonical NF-κB activation, mitogen-activated protein kinase (MAPK) signaling, and inflammasome-dependent inflammatory responses [26,27,28,29,30]. In addition to its inflammasome-associated activities, many biological functions of NLRP12 occur independently of inflammasome assembly, highlighting its broader role in coordinating innate immune signaling, maintaining immune homeostasis, and regulating context-dependent inflammatory responses [19,30]. More recently, NLRP12 has also been implicated in the regulation of programmed inflammatory cell death (PANoptosis), further expanding its functional repertoire beyond traditional inflammasome biology [31].

Recent studies have begun to reveal an unexpected connection between NLRP12 and skeletal integrity. NLRP12 expression is dynamically regulated during osteoclast differentiation, and genetic deletion of NLRP12 enhances osteoclastogenesis [32]. NLRP12-deficient mice exhibit increased osteoclast numbers, reduced bone mass, and exaggerated osteolytic responses, suggesting that NLRP12 functions as an important endogenous regulator of inflammatory bone remodeling [32]. Nevertheless, current evidence remains largely confined to osteoclasts and myeloid cells, whereas the roles of NLRP12 in osteoblasts, osteocytes, osteal macrophages (osteomacs), adaptive immunity, and the bone marrow immune microenvironment remain poorly understood.

Despite these emerging findings, the contribution of NLRP12 to osteoimmunology has not been comprehensively synthesized. Most studies have focused on systemic inflammation and host defense, whereas its broader roles in skeletal biology, inflammatory bone diseases, and human translation remain incompletely characterized [19]. In addition, important questions remain regarding the context-dependent functions of NLRP12, its relevance in human genetic disorders and inflammatory skeletal diseases, and its potential as a biomarker or therapeutic target. Recent advances in single-cell transcriptomics, spatial transcriptomics, quantitative proteomics, and metabolomics provide unprecedented opportunities to address these knowledge gaps and define the cell-specific regulatory networks governed by NLRP12 within the skeletal microenvironment [33,34].

In this narrative review, we synthesize current knowledge of the molecular biology, immunological functions, and emerging roles of NLRP12 in skeletal homeostasis and inflammatory bone diseases. Based on the available evidence, we propose that NLRP12 may represent a candidate osteoimmune checkpoint that integrates innate immune signaling with bone remodeling. We further discuss its translational potential, including human genetics, biomarker development, and future multi-omics approaches that may facilitate precision medicine strategies for inflammatory skeletal disorders. The current level of experimental and clinical evidence supporting each aspect of NLRP12 biology, together with the remaining knowledge gaps, is summarized in Supplemental Table S1.

## 2. Molecular Biology of NLRP12

Understanding the emerging role of NLRP12 in osteoimmunology first requires an appreciation of its molecular biology and immunoregulatory functions. The following sections summarize current knowledge of NLRP12 as an innate immune sensor and signaling regulator before discussing its roles in bone homeostasis [19,30,35,36].

### 2.1. NLRP12 as an Innate Immune Sensor

Innate immune receptors continuously monitor the intracellular and extracellular environment for conserved microbial products and endogenous danger signals that indicate infection or tissue injury [37]. As a member of the nucleotide-binding oligomerization domain (NOD)-like receptor (NLR) family, NLRP12 has long been classified as a cytosolic pattern recognition receptor (PRR) [19]. However, unlike well-characterized NLR family members such as NLRP3 or NOD2, the molecular mechanisms underlying NLRP12 activation remain incompletely understood. Although accumulating evidence supports a role for NLRP12 in sensing inflammatory perturbations, no definitive ligand has been identified to date, distinguishing NLRP12 from classical ligand-dependent PRRs [19].

Current evidence suggests that NLRP12 responds to a broad spectrum of pathogen-associated molecular patterns (PAMPs) and damage-associated molecular patterns (DAMPs), primarily through modulation of downstream inflammatory signaling rather than direct ligand recognition [18,19]. During bacterial infections, NLRP12 expression is induced following exposure to bacterial lipopolysaccharide (LPS), peptidoglycan, and other microbial components through Toll-like receptor (TLR)-mediated activation of NF-κB [38,39]. Viral and fungal pathogens have likewise been reported to alter NLRP12 expression, suggesting that NLRP12 functions as part of a secondary regulatory network that fine-tunes innate immune responses after PRR activation rather than acting as a primary microbial sensor [19,40]. This regulatory role enables NLRP12 to establish activation thresholds that prevent excessive inflammation while preserving effective host defense.

Beyond microbial stimuli, increasing evidence indicates that NLRP12 also responds to endogenous danger signals released during tissue damage. Sterile inflammatory conditions characterized by extracellular ATP, mitochondrial DNA, reactive oxygen species (ROS), uric acid crystals, and heme have all been associated with altered NLRP12 activity, although whether these molecules directly bind NLRP12 remains unknown [18,22]. Instead, NLRP12 is thought to sense perturbations in intracellular homeostasis or inflammatory signaling cascades triggered by these DAMPs. Such indirect sensing is consistent with the emerging concept that several NLR family members function as intracellular sentinels of cellular stress rather than conventional receptors for a single molecular ligand [17,18].

Recent studies have further expanded the functional repertoire of NLRP12 by implicating it in the regulation of programmed inflammatory cell death. In addition to its established role in restraining NF-κB and MAPK signaling, NLRP12 has been shown to participate in the assembly of the PANoptosome, a multiprotein complex that coordinates pyroptosis, apoptosis, and necroptosis, collectively termed PANoptosis [18,41]. Under specific inflammatory conditions, including heme-induced sterile inflammation and certain microbial infections, NLRP12 interacts with key PANoptotic components to regulate inflammatory cell death and cytokine production [18,42]. These findings suggest that NLRP12 possesses context-dependent functions that extend beyond suppression of inflammation and may, under appropriate conditions, promote host-protective inflammatory responses by facilitating coordinated cell death pathways.

Collectively, current evidence supports a model in which NLRP12 functions less as a classical ligand-specific receptor and more as a dynamic intracellular immune sensor that integrates microbial signals, tissue-derived danger cues, and cellular stress to fine-tune inflammatory responses. This unique sensing strategy may explain the diverse and sometimes opposing roles of NLRP12 reported across different tissues and disease settings. Within the skeletal microenvironment, where chronic exposure to inflammatory cytokines and damage-associated signals continuously influences bone remodeling, such context-dependent regulation positions NLRP12 as a potentially important modulator of osteoimmune homeostasis. The following sections discuss how these sensing properties converge on canonical inflammatory signaling pathways to regulate immune cell function and bone remodeling.

### 2.2. NLRP12 Regulation of NF-κB and MAPK Signaling

NLRP12 suppresses both NF-κB and MAPK signaling pathways, thereby constraining downstream inflammatory transcriptional responses. In the NF-κB axis, NLRP12 inhibits canonical signaling by attenuating IκB kinase activation, thereby reducing IκB phosphorylation and degradation, and limiting NF-κB nuclear translocation [35]. In parallel, NLRP12 regulates the non-canonical NF-κB pathway by promoting degradation of NF-κB–inducing kinase, a key upstream mediator required for p100 processing and RelB activation [32].

Beyond NF-κB, NLRP12 attenuates MAPK signaling, including ERK, JNK, and p38 pathways, which operate in parallel to regulate inflammatory transcriptional programs [35,43]. Deficiency of NLRP12 is associated with increased phosphorylation of these kinases, resulting in amplified inflammatory signaling and cytokine production, whereas NLRP12 overexpression suppresses MAPK activation in inflammatory cells, including fibroblast-like synoviocytes [44]. This positions NLRP12 as a coordinated regulator of convergent inflammatory signaling networks in osteoimmune contexts.

**Figure 1:**
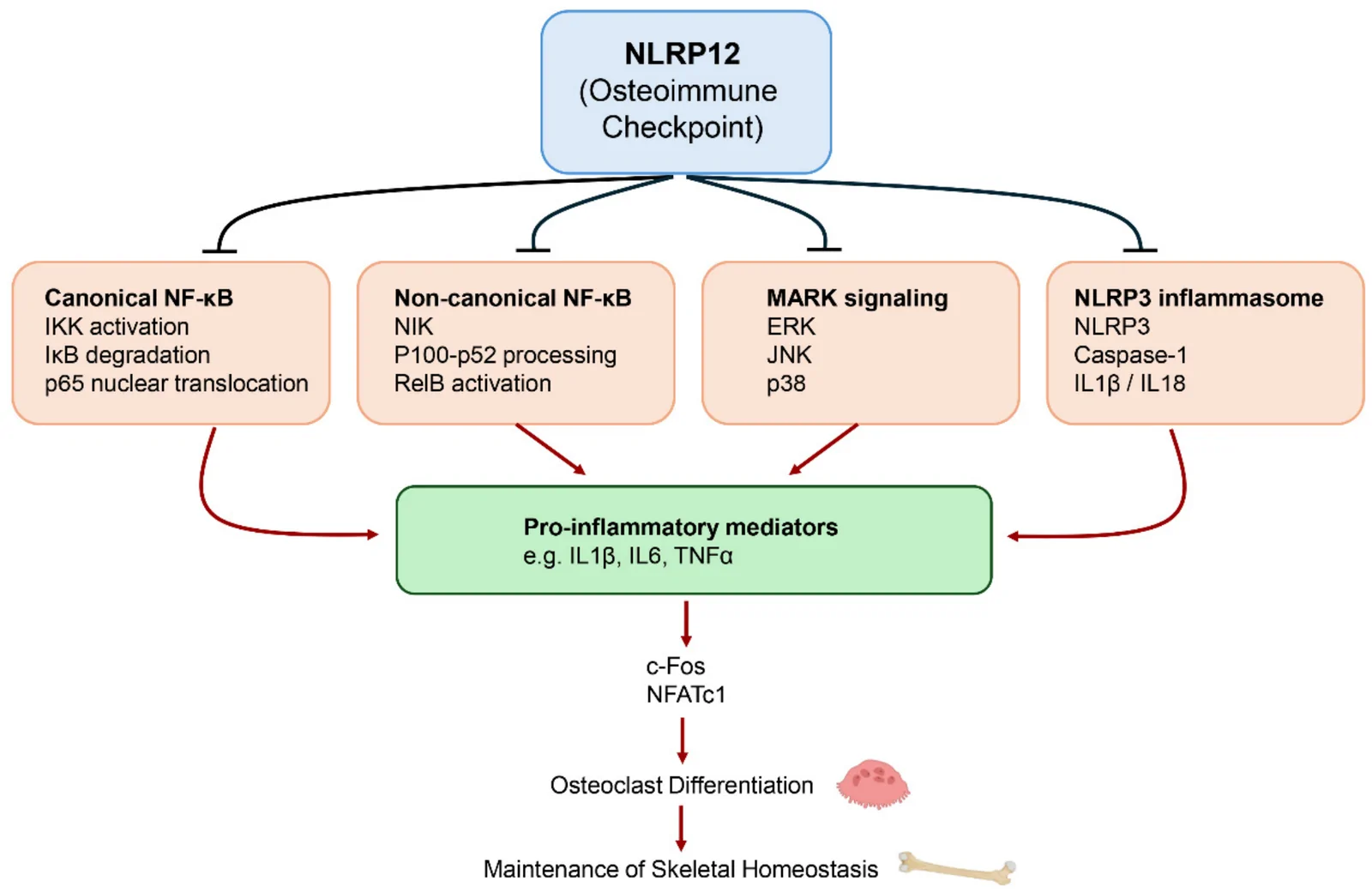
NLRP12 inhibits inflammatory signaling pathways to preserve osteoimmune homeostasis. NLRP12 functions as a negative regulator of multiple inflammatory signaling pathways that promote osteoclastogenesis and pathological bone resorption. NLRP12 suppresses canonical and non-canonical NF-κB signaling, MAPK activation, and NLRP3 inflammasome activity, thereby limiting the production of pro-inflammatory mediators, including TNF-α, IL-1β, and IL-6. Reduced inflammatory signaling attenuates the activation of the osteoclastogenic transcription factors c-Fos and NFATc1, leading to decreased osteoclast differentiation and bone resorption. Through coordinated inhibition of inflammatory and osteoclastogenic pathways, NLRP12 acts as an endogenous osteoimmune checkpoint that maintains skeletal homeostasis.

Collectively, these mechanisms position NLRP12 as an upstream regulator of inflammatory signaling networks that integrate NF-κB and MAPK activity to shape osteoimmune transcriptional output (Figure 1).

### 2.3. Beyond the Inflammasome: Inflammasome-Independent Functions of NLRP12

Although NLRP12 is frequently categorized as an inflammasome-associated receptor, current findings suggest that many of its biological functions are independent of classical inflammasome assembly [45]. In contrast to NLRP3, whose pathogenic effects are often mediated through caspase-1 activation and maturation of IL-1β and IL-18, NLRP12 primarily functions as a signaling regulator that restrains inflammatory responses upstream of cytokine production [19,45,46]. NLRP12’s anti-inflammatory activities extend beyond direct effects on innate immune cells and may shape the broader immune microenvironment that supports osteoclast differentiation [11,19,47]. These observations extend the functional role of NLRP12 beyond inflammasome regulation to broader control of inflammatory tone within osteoimmune environments.

### 2.4. Crosstalk Between NLRP12 and Inflammasome Signaling

Although NLRP12 is traditionally recognized as a negative regulator of inflammatory signaling, recent work demonstrates that its functions extend beyond modulation of NF-κB and MAPK pathways to include direct regulation of inflammasome activity. Among inflammasome-forming receptors, NLRP3 has received particular attention in osteoimmunology because of its established role in promoting inflammatory bone destruction [48].

Recent studies have revealed a previously unrecognized interaction between NLRP12 and NLRP3. Mechanistically, NLRP12 has been reported to associate with NLRP3 and interfere with inflammasome assembly, thereby limiting caspase-1 activation and downstream cytokine production [49]. This regulatory mechanism positions NLRP12 as an endogenous checkpoint that restrains excessive inflammasome activation and protects tissues from chronic inflammatory injury [19]. Disruption of NLRP12-mediated inhibition may create a permissive environment for inflammatory bone erosion.

The balance between NLRP12 and NLRP3 may therefore represent a key determinant of osteoimmune homeostasis. Under physiological conditions, NLRP12 constrains inflammatory responses and limits osteoclastogenic signaling, whereas loss of NLRP12 function may amplify NLRP3-dependent cytokine production and pathological bone resorption. This concept is particularly relevant in diseases such as rheumatoid arthritis, periodontitis, osteoporosis, and other osteolytic disorders in which inflammasome activation contributes to skeletal damage [50]. Future studies should investigate whether modulation of the NLRP12–NLRP3 axis can simultaneously suppress inflammation and preserve bone integrity, thereby providing a novel therapeutic strategy for inflammatory bone diseases (Figure 1).

### 2.5. Context-Dependent Biology of NLRP12

Although NLRP12 was initially characterized as a negative regulator of inflammation, accumulating evidence indicates that its biological functions are highly context-dependent and cannot be uniformly classified as anti-inflammatory [19,51]. Rather than acting as a constitutive inhibitor of immune activation, NLRP12 appears to function as a dynamic regulator that fine-tunes innate immune responses according to the nature of the stimulus, the affected tissue, the responding immune cell population, and the stage of disease [19,21,27]. This context dependency likely explains the seemingly contradictory observations reported across experimental models and highlights the importance of considering cellular and pathological environments when interpreting NLRP12 function.

Under conditions of sterile inflammation and chronic inflammatory diseases, NLRP12 predominantly functions as an anti-inflammatory regulator by suppressing canonical and non-canonical NF-κB signaling, limiting MAPK activation, and restraining inflammasome-dependent cytokine production [19]. Consequently, NLRP12 deficiency frequently results in excessive production of pro-inflammatory mediators, including TNF-α, interleukin (IL)-1β, IL-6, and IL-17, thereby exacerbating tissue inflammation and inflammatory bone loss [27,51]. These anti-inflammatory functions have been consistently demonstrated in experimental models of colitis, rheumatoid arthritis, obesity-associated inflammation, and periodontal disease, where NLRP12 limits pathological immune activation and promotes tissue homeostasis [52]. For example, Nlrp12-deficient mice develop more severe dextran sulfate sodium (DSS)-induced colitis characterized by enhanced NF-κB activation, increased inflammatory cytokine production, and impaired intestinal barrier function, supporting a protective role for NLRP12 in maintaining mucosal immune homeostasis [52].

In contrast, NLRP12 can also exert pro-inflammatory or host-protective functions during acute infection. Several studies have demonstrated that NLRP12 contributes to effective antimicrobial immunity by regulating leukocyte recruitment, neutrophil migration, cytokine production, and pathogen clearance [19,28]. More recently, NLRP12 has been identified as a component of the PANoptosome under specific inflammatory conditions, where it participates in the coordinated execution of pyroptosis, apoptosis, and necroptosis [18,53]. In these settings, NLRP12-mediated inflammatory cell death may facilitate elimination of infected or damaged cells and enhance host defense, illustrating that inflammatory signaling mediated by NLRP12 can be beneficial when appropriately controlled. Similarly, studies using bacterial infection models have shown that NLRP12 promotes neutrophil recruitment and effective pathogen clearance, demonstrating that transient NLRP12-dependent inflammatory responses can be advantageous during acute infection [54].

The biological functions of NLRP12 are also influenced by tissue-specific microenvironments. In mucosal tissues such as the intestine, NLRP12 plays a critical role in maintaining epithelial barrier integrity, regulating the intestinal microbiota, and preventing chronic inflammation [52,55]. Conversely, within the bone microenvironment, NLRP12 primarily limits inflammatory osteoclastogenesis and bone resorption rather than regulating epithelial barrier function, underscoring the tissue-specific nature of its biological activities [30,32]. These observations suggest that NLRP12 does not execute identical molecular programs across organs but instead adapts its regulatory functions to the local immune landscape and physiological demands of each tissue.

Cell-type specificity further contributes to the functional diversity of NLRP12. Most mechanistic studies have focused on macrophages, monocytes, dendritic cells, and neutrophils, where NLRP12 predominantly restrains inflammatory signaling pathways [22]. However, available data support that NLRP12 may influence adaptive immune responses indirectly by regulating cytokine networks that shape T helper cell differentiation, including the balance between regulatory T (Treg) cells and T helper 17 (Th17) cells [27,56]. Although direct evidence for cell-intrinsic regulation of adaptive lymphocytes remains limited, these observations suggest that NLRP12 may coordinate both innate and adaptive immunity through cell-specific regulatory networks.

Disease stage may represent an additional determinant of NLRP12 function. During the early phases of infection, transient inflammatory activation is essential for pathogen clearance and tissue protection, whereas persistent activation during chronic inflammation promotes tissue destruction and disease progression [57]. Accordingly, NLRP12 may facilitate protective immune responses during acute inflammatory challenges while functioning predominantly as an anti-inflammatory regulator during chronic inflammatory diseases [19,30]. This temporal regulation provides a plausible explanation for the distinct phenotypes observed across different experimental models and disease settings.

Collectively, current evidence supports a model in which NLRP12 functions as a context-dependent immune rheostat rather than a universal suppressor of inflammation (Figure 1). Its biological activity is determined by the integration of microbial stimuli, endogenous danger signals, tissue-specific microenvironments, immune cell identity, and disease stage. Recognizing this functional plasticity is essential for understanding the diverse roles of NLRP12 in osteoimmunology and will be critical for the rational development of NLRP12-targeted therapeutic strategies.

## 3. NLRP12 in Osteoimmunology

The field of osteoimmunology has established that bone remodeling is governed not only by intrinsic skeletal mechanisms but also by complex interactions among immune cells, inflammatory mediators, and bone-resident cells [58,59]. As a critical negative regulator of innate immune signaling, NLRP12 is uniquely positioned at the interface between immunity and bone remodeling. Experimental studies indicate that NLRP12 influences osteoclast differentiation directly while simultaneously shaping the inflammatory microenvironment that supports pathological bone resorption [32]. These observations support a broader role for NLRP12 as an osteoimmune checkpoint that coordinates immune activation with skeletal remodeling.

### 3.1. Regulation of Osteoclast Differentiation

NLRP12 functions as a negative regulator of osteoclast precursor sensitivity to inflammatory stimulation [32]. Its downregulation during differentiation suggests that reduced checkpoint control permits enhanced osteoclastogenic responsiveness. These findings suggest that NLRP12 sets the activation threshold for osteoclastogenic signaling rather than directly controlling differentiation programs [32].

### 3.2. Regulation of Osteoblasts, Osteocytes, and Osteomacs

Although current evidence firmly establishes NLRP12 as a regulator of osteoclast biology and myeloid cell function, considerably less is known regarding its roles in bone-forming and bone-resident cells. Bone remodeling is a highly coordinated process requiring continuous communication among osteoclasts, osteoblasts, osteocytes, and osteal macrophages (osteomacs) [60]. While direct evidence for NLRP12 function in these cellular compartments remains limited, its established ability to regulate inflammatory signaling suggests that NLRP12 may indirectly influence multiple aspects of skeletal homeostasis beyond osteoclastogenesis.

Osteoblasts are responsible for bone matrix synthesis and mineralization and are highly sensitive to inflammatory signals within the bone microenvironment [61]. Chronic exposure to pro-inflammatory cytokines suppresses osteoblast differentiation, impairs bone formation, and disrupts the balance between RANKL and osteoprotegerin (OPG), thereby indirectly promoting osteoclastogenesis [62]. Although NLRP12 expression has not been systematically characterized in osteoblasts, several inflammatory pathways negatively regulated by NLRP12, including NF-κB and MAPK signaling, are well-established inhibitors of osteoblast differentiation and function [32]. These observations raise the possibility that NLRP12 may indirectly support osteogenesis by maintaining an anti-inflammatory microenvironment that favors bone formation. Future studies examining osteoblast-specific expression and function of NLRP12 are needed to determine whether these effects are mediated through cell-intrinsic mechanisms or through regulation of the surrounding immune milieu.

Osteocytes, the most abundant cells within bone, serve as the principal mechanosensors of the skeleton and orchestrate bone remodeling through the coordinated production of RANKL and OPG in response to mechanical and inflammatory stimuli [63]. Chronic inflammation stimulates osteocyte-derived RANKL expression, thereby enhancing osteoclast differentiation and pathological bone resorption [8]. Given its established role as a negative regulator of inflammatory signaling, NLRP12 may function to limit inflammatory activation within osteocytes and preserve bone homeostasis [64]. In addition to inflammatory regulation, NLRP12 may also participate in mechanotransduction, as mechanical loading activates NF-κB and MAPK signaling pathways that are negatively regulated by NLRP12 [65,66]. However, whether NLRP12 is expressed in osteocytes under physiological or pathological conditions remains unknown, representing an important gap in our understanding of osteoimmune regulation.

Another potentially important but understudied cellular target of NLRP12 is the osteal macrophage (osteomac), a specialized population of resident macrophages located along bone surfaces that supports skeletal maintenance [67]. Unlike inflammatory macrophages that promote osteoclastogenesis through cytokine production, osteomacs facilitate osteoblast survival, matrix mineralization, and fracture repair [68,69]. Because NLRP12 is highly expressed in myeloid cells and regulates macrophage activation, osteomacs represent a plausible cellular compartment through which NLRP12 may influence bone formation and tissue repair. By restraining excessive inflammatory activation while preserving tissue-supportive macrophage functions, NLRP12 may contribute to maintaining an anabolic bone microenvironment.

Although these observations support broader roles for NLRP12 in skeletal integrity, direct evidence in osteoblasts, osteocytes, and osteomacs remains limited. The following section expands this concept by examining how NLRP12 shapes the broader bone marrow immune microenvironment.

### 3.3. NLRP12 in the Bone Marrow Immune Microenvironment

Bone remodeling occurs within a highly specialized immune microenvironment composed of myeloid and lymphoid populations that collectively regulate osteoclastogenesis [70]. Although the direct skeletal functions of NLRP12 remain incompletely characterized, its established roles in multiple immune cell populations suggest that it may exert broader control over the osteoclastogenic niche.

Macrophages

Macrophages play central roles in both innate immunity and bone remodeling through the production of osteoclastogenic cytokines such as TNF-α, IL-1β, and IL-6 [4,71,72]. Accordingly, NLRP12 has been investigated primarily in myeloid cells, particularly bone marrow-derived macrophages, where it regulates inflammatory signaling pathways and cytokine responses [19,21,32,35,73,74]. Loss of NLRP12 enhances NF-κB activation and pro-inflammatory cytokine production, whereas its expression constrains inflammatory signaling and reduces osteoclastogenic cytokine output within the bone marrow niche [19,32]. Emerging evidence also suggests that NLRP12 may influence macrophage polarization [75,76]. By restraining pro-inflammatory responses, NLRP12 may facilitate resolution of inflammation and preservation of tissue homeostasis, although its specific role in osteal macrophages (osteomacs) remains largely unexplored.

Dendritic Cells

Dendritic cells contribute to osteoimmune regulation through antigen presentation, cytokine secretion, and interactions with T cells [77]. NLRP12 has been shown to regulate dendritic cell migration and inflammatory responses, suggesting that it may influence immune activation within skeletal tissues [24,78]. Whether NLRP12-dependent regulation of dendritic cells directly affects osteoclastogenesis remains unknown and represents an important area for future investigation.

Neutrophils

Neutrophils are increasingly recognized as contributors to inflammatory bone loss through the release of cytokines, reactive oxygen species, and neutrophil extracellular traps (NETs) [79]. NLRP12 is expressed in neutrophils and regulates their inflammatory activity and trafficking [80]. Given the emerging role of neutrophils in rheumatoid arthritis, periodontitis, and other osteolytic diseases, NLRP12 may indirectly modulate skeletal pathology through regulation of neutrophil-mediated inflammation [81].

Myeloid Precursors

Osteoclasts arise from myeloid progenitor populations that are highly responsive to inflammatory signals [82]. Because NLRP12 is expressed throughout the myeloid lineage, it may influence the developmental programming of osteoclast precursors before RANKL exposure [32]. This possibility raises the intriguing concept that NLRP12 regulates not only mature inflammatory responses but also the intrinsic osteoclastogenic potential of myeloid progenitors. Future studies utilizing single-cell transcriptomics and lineage-tracing approaches may help define these developmental functions.

Collectively, these observations suggest that NLRP12 may regulate bone remodeling at multiple levels by simultaneously controlling inflammatory cytokine production, immune-cell activation, and osteoclast precursor differentiation. Such integrated regulation is consistent with the proposed role of NLRP12 as a central osteoimmune checkpoint.

### 3.4. Integration of Innate and Adaptive Osteoimmunity

Bone remodeling is regulated through coordinated interactions between innate and adaptive immune cells, a process that forms the foundation of osteoimmunology [83]. While the role of NLRP12 in innate immune cells, particularly macrophages and dendritic cells, has been extensively investigated, its influence on adaptive immune responses within the skeletal microenvironment remains less well defined [19]. Nevertheless, growing evidence suggests that innate immune signaling profoundly shapes T-cell differentiation and function, raising the possibility that NLRP12 may indirectly regulate adaptive osteoimmune responses through its effects on inflammatory cytokine networks [84].

Among adaptive immune populations, the balance between Treg cells and Th17 cells is recognized as a critical determinant of inflammatory bone remodeling [85]. Treg cells suppress osteoclastogenesis through the production of anti-inflammatory cytokines, including interleukin (IL)-10 and transforming growth factor-β (TGF-β), whereas Th17 cells promote bone resorption by secreting IL-17, RANKL, and other osteoclastogenic mediators that stimulate osteoclast differentiation and activation [86]. Disruption of the Treg/Th17 equilibrium has been implicated in numerous inflammatory skeletal disorders, including rheumatoid arthritis, osteoporosis, and periodontitis, highlighting this axis as an important regulator of pathological bone loss [87].

Although direct evidence linking NLRP12 to Treg or Th17 differentiation is still emerging, several observations support a potential functional connection. NLRP12 negatively regulates the production of inflammatory cytokines, including IL-1β, IL-6, and TNF-α, through suppression of NF-κB and MAPK signaling [51]. These cytokines are well-established drivers of Th17 polarization while simultaneously impairing Treg stability and function [87]. Consequently, NLRP12-mediated restraint of innate inflammatory signaling may indirectly favor a more balanced adaptive immune response by limiting the cytokine milieu that promotes osteoclastogenic Th17 immunity. Conversely, loss of NLRP12 may amplify inflammatory cytokine production, thereby creating conditions that favor Th17 expansion and enhanced bone resorption [56].

Whether NLRP12 also exerts cell-intrinsic functions within adaptive immune cells remains an important unanswered question. Future studies integrating single-cell transcriptomics, conditional knockout mouse models, and immune profiling of inflammatory bone diseases will be essential to determine whether NLRP12 directly regulates T-cell differentiation or primarily influences adaptive immunity through myeloid cell-mediated cytokine networks [88]. Elucidating these mechanisms will provide a more comprehensive understanding of how innate and adaptive immune pathways converge to regulate bone homeostasis and may further strengthen the emerging concept of NLRP12 as a candidate osteoimmune checkpoint. Overall, current evidence supports an emerging role for NLRP12 in coordinating innate and adaptive osteoimmune responses, although the underlying mechanisms require further experimental validation.

## 4. Human Translation and Clinical Relevance

Although mechanistic studies have substantially advanced our understanding of NLRP12 biology, the vast majority of current evidence has been generated using murine models of inflammation and genetic knockout systems. Translation of these findings to human skeletal diseases remains at an early stage, and significant gaps persist regarding the clinical relevance of NLRP12 in osteoimmunology. Bridging this translational gap will require integration of human genetics, patient-derived biospecimens, and emerging multi-omics technologies to determine whether NLRP12 represents a clinically actionable biomarker or therapeutic target.

### 4.1. Human Genetics and NLRP12-Associated Disease

The strongest evidence supporting the physiological importance of NLRP12 in humans comes from studies of NLRP12-associated autoinflammatory disease (NLRP12-AID), also referred to as familial cold autoinflammatory syndrome type 2 (FCAS2) [89]. Germline variants in NLRP12 have been associated with recurrent fever episodes, arthritis, arthralgia, urticaria, and systemic inflammation, demonstrating that disruption of NLRP12 signaling can profoundly alter immune homeostasis [89,90]. However, unlike highly penetrant monogenic autoinflammatory disorders, NLRP12-associated disease exhibits considerable phenotypic heterogeneity, incomplete penetrance, and variable clinical severity, suggesting that additional genetic, epigenetic, and environmental factors influence disease expression [91].

Beyond rare pathogenic variants, several single-nucleotide polymorphisms (SNPs) and sequence variants within the NLRP12 locus have been reported in inflammatory and autoimmune disorders [92]. Although their functional significance remains incompletely understood, these findings raise the possibility that naturally occurring variation in NLRP12 may contribute to inter-individual differences in inflammatory responses and susceptibility to immune-mediated diseases [19]. To date, however, the relationship between NLRP12 polymorphisms and bone remodeling, osteoporosis, rheumatoid arthritis progression, or periodontal bone loss has not been systematically investigated, representing an important area for future clinical research.

### 4.2. Translational Challenges: From Mouse Models to Human Disease

Most mechanistic insights into NLRP12 function have been derived from constitutive knockout mice, conditional genetic models, or in vitro studies using murine macrophages [24,35]. While these models have established NLRP12 as an important regulator of NF-κB, MAPK, inflammasome signaling, and inflammatory bone loss, direct extrapolation to human disease should be approached with caution. Species-specific differences in immune regulation, genetic background, microbiota composition, and disease pathogenesis may influence NLRP12 function and contribute to discrepancies between experimental models and clinical observations.

Furthermore, current knowledge of NLRP12 expression in human skeletal tissues remains limited. Whether NLRP12 is differentially expressed in osteoclasts, osteoblasts, osteocytes, osteal macrophages, or bone marrow immune populations during inflammatory bone diseases has yet to be comprehensively determined. Future studies employing human bone biopsies, synovial tissue, periodontal lesions, and single-cell transcriptomic analyses will be essential for validating experimental findings and defining the cell-specific functions of NLRP12 within the human skeletal microenvironment.

### 4.3. Biomarker Potential and Precision Medicine

Beyond its mechanistic role, NLRP12 has emerged as a promising biomarker candidate for inflammatory skeletal diseases, although current evidence remains at the preclinical and translational research stage. At present, the strongest evidence supports the use of NLRP12 as a marker of innate immune activation and inflammatory signaling rather than as a validated diagnostic or prognostic biomarker. Quantification of NLRP12 expression in peripheral blood mononuclear cells (PBMCs), circulating monocytes, or inflamed tissue specimens may provide insight into systemic immune activation and inflammatory status [21]. Likewise, integrating NLRP12 expression with circulating inflammatory mediators, such as IL-1β, IL-6, TNF-α, RANKL, OPG, C-terminal telopeptide of type I collagen (CTX), and procollagen type I N-terminal propeptide (P1NP), may improve assessment of bone remodeling dynamics and disease activity [45].

Future studies should determine whether NLRP12 expression levels or genetic variants possess prognostic or patient-stratification value in inflammatory skeletal diseases. For example, longitudinal studies evaluating NLRP12 expression or genetic variants in patients with osteoporosis, rheumatoid arthritis, periodontitis, or inflammatory osteolysis could determine whether altered NLRP12 signaling predicts disease progression, fracture risk, treatment response, or recurrence following therapy. Integration of genomic, transcriptomic, and clinical datasets may further facilitate identification of patient subgroups most likely to benefit from therapies targeting inflammatory pathways regulated by NLRP12 [23].

Collectively, current evidence supports the biological relevance of NLRP12 in human immune regulation but remains insufficient to establish its clinical utility in osteoimmune diseases. A practical translational pathway would include: (i) validating NLRP12 expression in PBMCs and inflamed skeletal tissues from well-characterized patient cohorts; (ii) correlating NLRP12 expression or genetic variants with clinical outcomes, including bone mineral density, CTX, P1NP, fracture risk, and treatment response; and (iii) integrating these data with multi-omics analyses to evaluate whether NLRP12 provides independent diagnostic, prognostic, or patient-stratification value. These studies will determine whether NLRP12 can ultimately be translated into a clinically useful biomarker and precision medicine indicator for inflammatory skeletal disorders.

## 5. NLRP12 in Bone Diseases

Although most mechanistic studies have been performed in experimental models, growing evidence suggests that dysregulated NLRP12 signaling contributes to multiple inflammatory skeletal diseases. The following sections summarize current evidence across major bone disorders.

### 5.1. Osteoporosis

Osteoporosis is the most common metabolic bone disease and is characterized by reduced bone mass and increased fracture risk [93]. While aging, hormonal changes, and mechanical factors contribute to disease progression, chronic low-grade inflammation has emerged as an important driver of bone loss [94].

Current evidence suggests that NLRP12 functions as an endogenous suppressor of osteoclastogenesis in this context. NLRP12-deficient mice exhibit increased osteoclast formation and reduced bone mass, supporting a physiological role for NLRP12 in maintaining bone physiology [32]. These findings suggest that reduced NLRP12 activity may contribute to age-associated bone loss. Although direct clinical studies examining NLRP12 in osteoporosis are currently lacking, the established links between NLRP12, inflammatory signaling, and osteoclast differentiation suggest that this pathway may represent a previously underappreciated regulator of skeletal aging.

### 5.2. Rheumatoid Arthritis

Rheumatoid arthritis (RA) is a chronic autoimmune disease characterized by persistent synovial inflammation, progressive cartilage destruction, and focal bone erosion [95]. Osteoclasts are the primary mediators of bone damage in RA, and their differentiation is driven by abundant pro-inflammatory cytokines within the inflamed synovium [96].

Recent studies have identified NLRP12 as an important negative regulator of inflammatory responses in RA-associated fibroblast-like synoviocytes (FLSs) [44]. Reduced NLRP12 expression enhances inflammatory signaling and indirectly promotes osteoclast-mediated tissue damage [44]. Conversely, restoration of NLRP12 expression suppresses inflammatory signaling and attenuates cytokine production. These findings suggest a protective role for NLRP12 in limiting structural joint damage.

### 5.3. Periodontitis and Apical Periodontitis

Periodontitis and apical periodontitis are chronic inflammatory diseases characterized by local destruction of alveolar bone in response to microbial infection [97]. Persistent activation of innate immune pathways leads to excessive production of inflammatory cytokines and recruitment of osteoclasts, resulting in progressive bone loss surrounding teeth [98].

Among skeletal disorders, apical periodontitis provides some of the strongest evidence supporting a protective role for NLRP12 in inflammatory bone disease. Experimental studies have demonstrated that NLRP12 deficiency exacerbates inflammatory bone destruction and increases osteoclast activity within periapical lesions [99]. These findings highlight the importance of NLRP12 in controlling infection-induced osteolysis and support the concept that NLRP12 functions as a local osteoimmune checkpoint within inflamed skeletal tissues. Given the shared inflammatory pathways underlying periodontitis and apical periodontitis, similar protective mechanisms may operate across a broader spectrum of oral inflammatory bone diseases.

### 5.4. Emerging Skeletal Disorders

Although direct evidence remains limited, the biological functions of NLRP12 suggest potential relevance to several additional skeletal disorders characterized by inflammation-driven bone remodeling.

Fracture Healing

Successful fracture repair requires tightly coordinated inflammatory and regenerative responses. While early inflammation is necessary for recruitment of reparative cells, excessive or prolonged inflammation can impair bone regeneration [100]. Through its ability to limit inflammatory signaling while preserving tissue homeostasis, NLRP12 may contribute to the resolution phase of fracture healing [101]. However, direct experimental evidence linking NLRP12 to skeletal repair remains unavailable.

Bone Metastasis

Bone metastases generate a highly inflammatory microenvironment that promotes osteoclast activation and pathological bone destruction. Given its broad anti-inflammatory functions, NLRP12 may influence tumor-associated osteolysis by modulating immune-cell activation and osteoclastogenic signaling [30,102]. Although speculative, this possibility warrants investigation in future studies of cancer-induced bone disease.

Current evidence supports a regulatory role for NLRP12 in inflammatory bone pathology across multiple skeletal contexts. While the strongest experimental support currently exists in osteoporosis-related bone remodeling, rheumatoid arthritis, and apical periodontitis, current findings suggest that NLRP12 may influence a much broader spectrum of skeletal disorders [32,99]. These observations further support the concept of NLRP12 as an osteoimmune checkpoint whose dysfunction may contribute to pathological bone destruction across diverse disease settings.

## 6. Emerging Concepts and Future Perspectives

**Figure 2:**
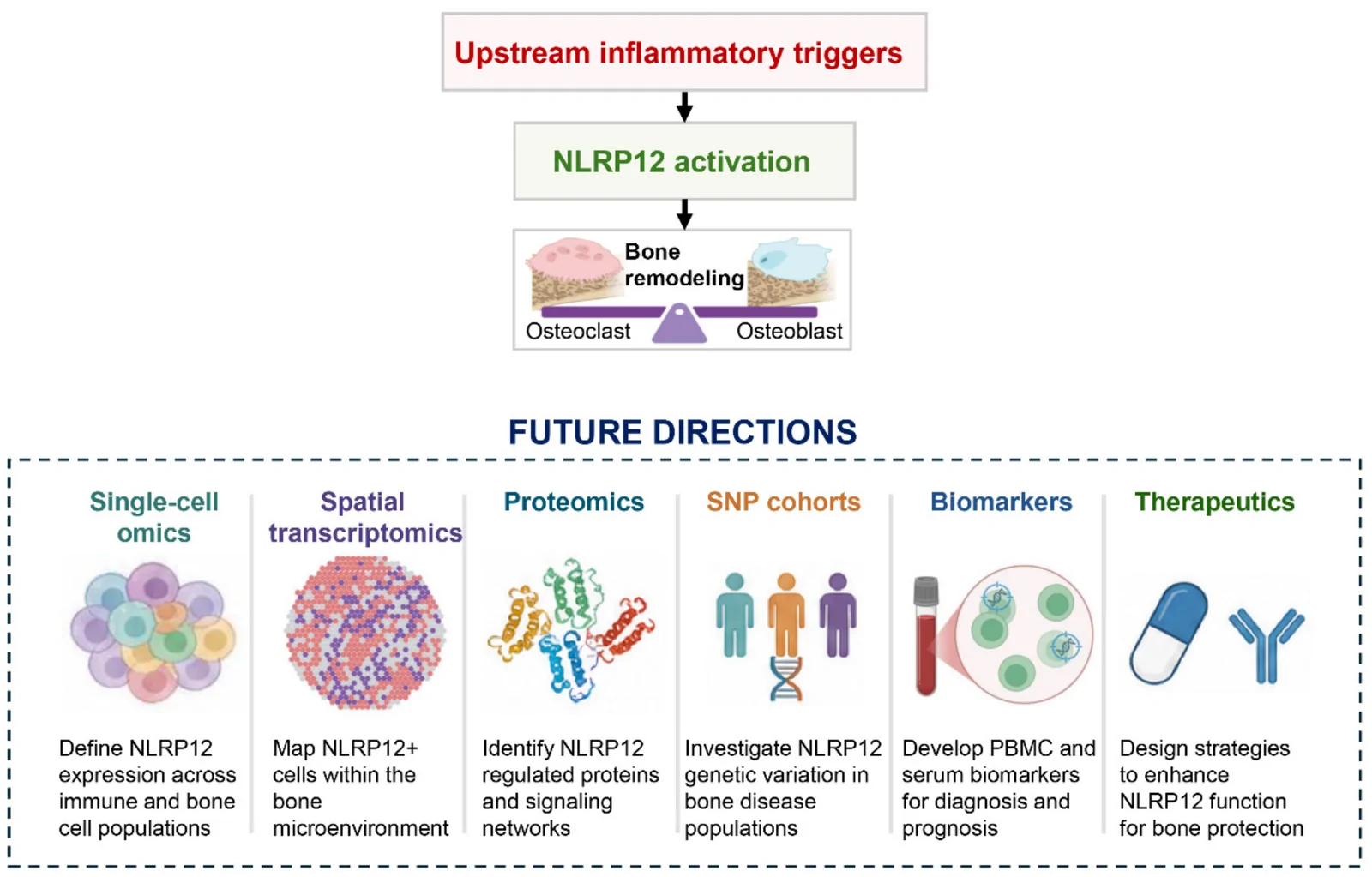
Translational framework and future horizons for NLRP12 in osteoimmunology. Upstream inflammatory triggers stimulate NLRP12 activation, which serves as a critical molecular checkpoint governing downstream bone remodeling by modulating the functional balance between osteoclasts and osteoblasts. Translating these mechanistic insights into precision osteoimmune care relies on key integrated approaches: single-cell omics and spatial transcriptomics to define cellular heterogeneity and map high-resolution microenvironmental niches; proteomics to characterize underlying regulatory signaling networks; and SNP cohorts to identify genetic variants linked to disease susceptibility. Ultimately, these pillars feed directly into biomarker discovery and targeted therapeutics to advance clinical diagnostics and personalized bone-protective interventions.

Despite growing evidence supporting a role for NLRP12 in regulating inflammatory signaling and osteoclast differentiation, many aspects of its function in skeletal biology remain poorly understood. Most studies to date have focused on the anti-inflammatory properties of NLRP12 in innate immune cells, whereas its broader contributions to bone homeostasis have received comparatively little attention. Emerging advances in immunometabolism, aging biology, and osteoimmunology provide new opportunities to understand how NLRP12 may integrate immune and skeletal responses under physiological and pathological conditions (Figure 2).

### 6.1. Emerging Multi-Omics and Immunometabolic Reprogramming

Recent advances in multi-omics technologies have transformed our understanding of osteoimmunology by enabling high-resolution characterization of immune cell heterogeneity, molecular signaling networks, and metabolic reprogramming within the skeletal microenvironment. Single-cell RNA sequencing (scRNA-seq), spatial transcriptomics, quantitative proteomics, and metabolomics now permit comprehensive analysis of cell-specific transcriptional states, protein expression, metabolic pathways, and intercellular communication during inflammatory bone remodeling [33]. Collectively, these approaches provide unprecedented opportunities to elucidate how NLRP12 coordinates immune regulation across diverse cellular populations and disease contexts. Although direct multi-omics studies of NLRP12 in skeletal diseases remain limited, these technologies are expected to substantially accelerate mechanistic and translational investigations in osteoimmunology [103,104].

Single-cell RNA sequencing has revealed remarkable heterogeneity within bone marrow immune populations, identifying distinct macrophage, osteoclast precursor, dendritic cell, and lymphocyte subsets that differentially regulate bone remodeling [105]. Integration of scRNA-seq with spatial transcriptomics further enables mapping of immune cell localization and cellular interactions within the bone marrow niche, thereby providing insight into how inflammatory signaling is coordinated in situ [106]. Future application of these technologies to NLRP12-deficient models or human inflammatory bone diseases could define the cell-specific expression patterns of NLRP12, identify downstream transcriptional programs, and clarify how NLRP12 regulates communication among osteoclasts, osteoblasts, osteocytes, osteomacs, and infiltrating immune cells.

Complementary proteomic and metabolomic analyses provide an additional layer of biological information that cannot be inferred from transcriptomic data alone. Quantitative proteomics enables comprehensive characterization of inflammatory signaling networks, post-translational modifications, and cytokine responses regulated by NLRP12, whereas metabolomics identifies metabolic intermediates that govern immune cell activation and osteoclast differentiation [107,108]. Integration of these datasets with transcriptomic analyses will facilitate systems-level reconstruction of NLRP12-regulated signaling networks and may identify novel biomarkers or therapeutic targets for inflammatory skeletal diseases.

Recent advances in immunometabolism have further highlighted the importance of cellular metabolic programs in shaping immune-cell activation and osteoimmune responses [109]. Rather than serving solely as an energy source, metabolic pathways actively regulate inflammatory signaling, cytokine production, and cell fate decisions [110]. Because NLRP12 functions as a critical regulator of innate immune activation, an emerging concept is that NLRP12 may influence bone remodeling through modulation of immunometabolic reprogramming.

Activated macrophages and osteoclast precursors undergo profound metabolic changes characterized by increased glycolysis, enhanced mitochondrial activity, and altered nutrient sensing [111]. These adaptations support the energetic demands of inflammatory responses and osteoclast differentiation [112]. Metabolic intermediates such as succinate have emerged as important signaling molecules that promote inflammatory cytokine production and osteoclastogenesis through stabilization of hypoxia-inducible factor-1α (HIF-1α) [113]. Increased succinate accumulation has been associated with inflammatory bone destruction and enhanced osteoclast activity, underscoring the tight coupling between metabolic regulation and bone remodeling [114].

Several nutrient-sensing pathways may intersect with NLRP12 function. The mammalian target of rapamycin (mTOR) pathway promotes anabolic metabolism and inflammatory activation, whereas AMP-activated protein kinase (AMPK) acts as a metabolic checkpoint that restrains inflammation and supports cellular homeostasis [115]. Dysregulation of the mTOR–AMPK axis has been implicated in osteoporosis, rheumatoid arthritis, and other osteolytic disorders. Although direct evidence remains limited, NLRP12-mediated suppression of inflammatory signaling may influence these metabolic pathways and thereby regulate osteoclastogenic potential [19,116,117].

Mitochondrial metabolism provides another potential point of convergence. Mitochondrial dysfunction and excessive reactive oxygen species production contribute to inflammatory activation and osteoclast differentiation [118]. By restraining inflammatory signaling and maintaining immune homeostasis, NLRP12 may indirectly limit the metabolic conditions that favor pathological bone resorption [40]. Looking forward, integrated multi-omics strategies combining scRNA-seq, spatial transcriptomics, quantitative proteomics, metabolomics, and functional metabolic analyses will be instrumental in defining the molecular circuits governed by NLRP12 during inflammatory bone remodeling. Such systems-level investigations may establish NLRP12 as a critical molecular interface linking inflammation, cellular metabolism, and skeletal integrity while facilitating the discovery of biomarkers and therapeutic targets for precision osteoimmunology.

### 6.2. NLRP12 and the Gut–Bone Axis

The gut microbiota has emerged as a major regulator of systemic immunity and skeletal health, giving rise to the concept of the gut–bone axis [119]. Alterations in microbial composition influence immune-cell activation, cytokine production, nutrient absorption, and bone remodeling, whereas dysbiosis has been linked to osteoporosis, inflammatory arthritis, and other disorders characterized by pathological bone loss [120].

Among members of the NLR family, NLRP12 plays a particularly important role in maintaining intestinal homeostasis [121]. Experimental studies have demonstrated that NLRP12 regulates gut microbial composition, suppresses intestinal inflammation, and protects against dysbiosis-associated disease [52,121]. Loss of NLRP12 results in exaggerated inflammatory responses within the gastrointestinal tract, accompanied by alterations in microbial communities that promote chronic immune activation [84]. These observations suggest that NLRP12 functions as an important mediator of host–microbiota interactions.

Intestinal inflammation can influence skeletal remodeling through systemic dissemination of inflammatory cytokines and microbial metabolites [122]. Elevated circulating levels of TNF-α, IL-1β, and IL-6 promote osteoclastogenesis and contribute to bone loss, while microbiota-derived metabolites can directly affect immune and skeletal cell function [123,124]. By preserving intestinal immune homeostasis, NLRP12 may indirectly regulate osteoclastogenic signaling at distant skeletal sites.

This framework raises the intriguing possibility that NLRP12 contributes to bone homeostasis through mechanisms extending beyond the bone marrow microenvironment. In addition to its direct effects on immune cells and osteoclast precursors, NLRP12 may influence skeletal remodeling through regulation of microbial ecology and systemic inflammatory tone. Future studies integrating microbiome profiling, metabolomics, and skeletal phenotyping will be essential to determine whether the gut–bone axis represents an important component of the proposed NLRP12 osteoimmune checkpoint framework.

### 6.3. Toward an NLRP12 Osteoimmune Checkpoint Framework

**Figure 3:**
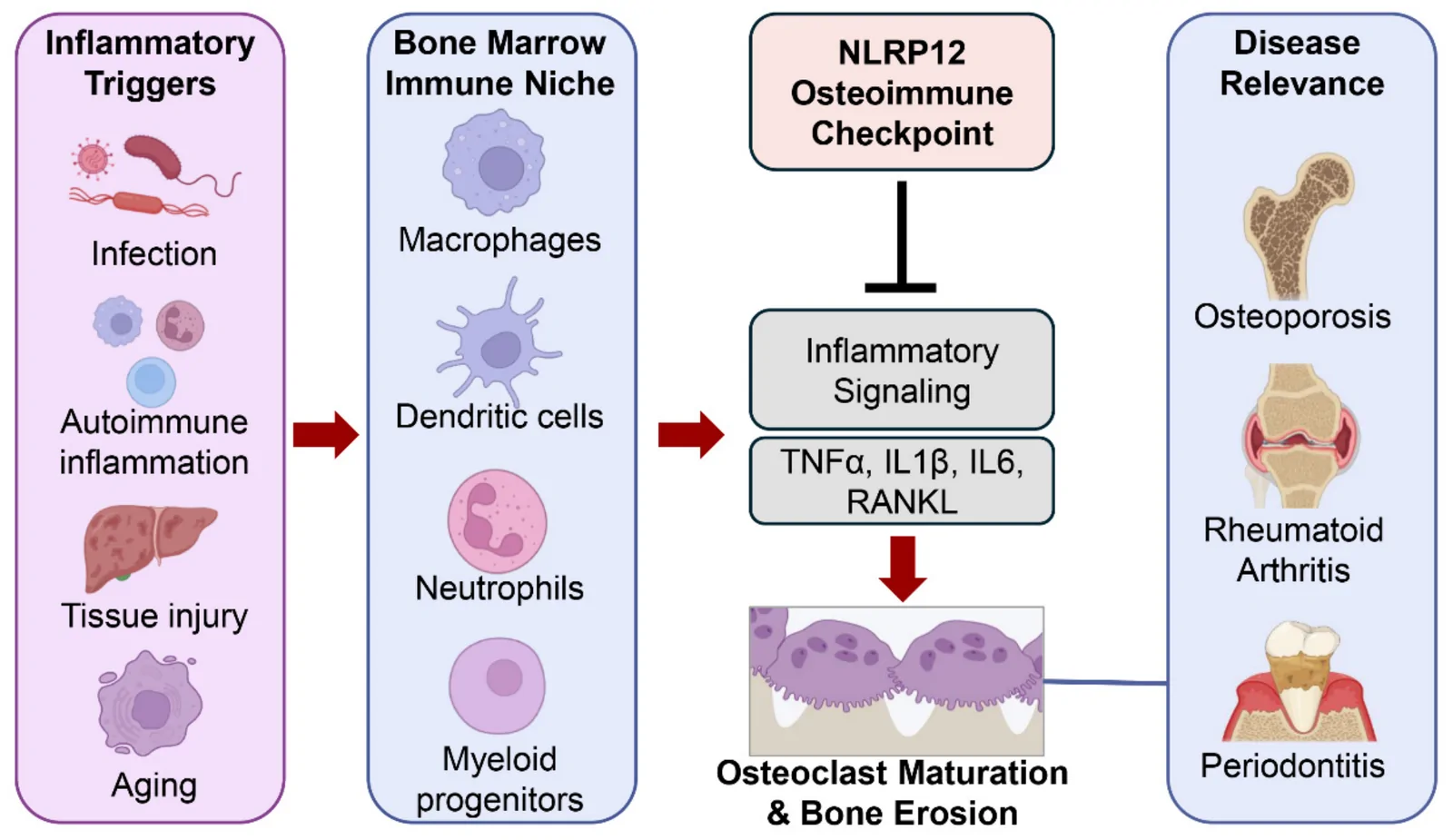
The NLRP12 osteoimmune checkpoint framework in inflammatory bone remodeling. NLRP12 is proposed to function as a central osteoimmune checkpoint that coordinates immune activation and skeletal remodeling. Inflammatory stimuli, including microbial products, tissue injury, autoimmune responses, and aging-associated inflammation, activate immune cells within the bone marrow niche and promote the production of osteoclastogenic mediators such as TNF-α, IL-1β, IL-6, and RANKL. These signals drive osteoclast differentiation and pathological bone resorption. NLRP12 counteracts these responses by suppressing inflammatory signaling pathways and limiting osteoclastogenic activity, thereby maintaining skeletal homeostasis. Disruption of this regulatory network may contribute to inflammatory bone disorders, including osteoporosis, rheumatoid arthritis, and periodontitis.

The evidence summarized throughout this review supports a conceptual framework in which NLRP12 may function as a candidate osteoimmune checkpoint that integrates inflammatory signaling with skeletal remodeling (Figure 3) [19]. Unlike classical immune checkpoint molecules that primarily regulate adaptive immunity, NLRP12 appears to operate predominantly within the innate immune system by fine-tuning inflammatory signaling pathways that influence osteoclastogenesis, immune-cell activation, and bone homeostasis [19,32]. Rather than functioning as a universal suppressor of inflammation, NLRP12 maintains immune equilibrium, thereby limiting excessive inflammatory responses while preserving protective host defense [19,40]. This emerging model provides a unifying framework that integrates the diverse biological functions of NLRP12 described across inflammatory and skeletal diseases.

Within this framework, PAMPs, DAMPs, inflammatory cytokines, tissue injury, and chronic immune activation stimulate intracellular signaling pathways, including NF-κB, MAPK, and inflammasome-associated cascades, resulting in the production of pro-inflammatory mediators that promote osteoclast differentiation and pathological bone resorption [125,126]. NLRP12 functions as a regulatory node within these signaling networks by restraining excessive inflammatory activation, modulating cytokine production, and shaping the activity of macrophages, dendritic cells, neutrophils, and other myeloid populations that collectively define the bone marrow immune microenvironment. Through these coordinated actions, NLRP12 may indirectly influence osteoblasts, osteocytes, osteomacs, and adaptive immune responses, thereby contributing to the maintenance of osteoimmune homeostasis.

Importantly, the current evidence indicates that the influence of NLRP12 extends beyond regulation of individual cell populations. Emerging evidence suggests that NLRP12 integrates inflammatory signaling, immunometabolism, microbiota–host interactions, and multicellular communication within the skeletal niche [19,30]. Advances in single-cell transcriptomics, spatial transcriptomics, quantitative proteomics, and metabolomics now provide unprecedented opportunities to define the cell-specific functions and regulatory networks governed by NLRP12 at systems-level resolution [127,128]. These technologies will be instrumental in determining whether NLRP12 coordinates multicellular osteoimmune interactions that cannot be appreciated through conventional bulk analyses.

Despite substantial progress in elucidating the immunoregulatory functions of NLRP12, several important questions remain unanswered. The upstream mechanisms governing NLRP12 activation; its cell-specific functions within osteoblasts, osteocytes, osteomacs, and adaptive immune cells; and the translational relevance of human NLRP12 genetic variation require further investigation. Likewise, validation of NLRP12 as a prognostic biomarker or therapeutic target will require well-designed clinical studies integrating human genetics, patient-derived biospecimens, and multi-omics analyses. Addressing these challenges will determine whether NLRP12 ultimately fulfills the criteria of a bona fide osteoimmune checkpoint or is more appropriately viewed as a pivotal regulator of inflammatory bone remodeling. Regardless, the accumulating evidence highlights NLRP12 as a promising molecular interface linking innate immunity and skeletal biology, providing a strong rationale for continued investigation into its biological functions and therapeutic potential.

## 7. Conclusions

NLRP12 is emerging as an important regulator of the osteoimmune interface. Through suppression of NF-κB, MAPK, and pro-inflammatory signaling pathways, NLRP12 limits osteoclastogenesis and protects against pathological bone resorption. While current evidence remains limited compared with other NLR family members, available studies consistently support a protective role for NLRP12 in inflammatory bone diseases. We propose the NLRP12 osteoimmune checkpoint framework as a unifying model that integrates immune regulation, osteoclast biology, and skeletal homeostasis. Continued investigation of this pathway may reveal new therapeutic opportunities for osteoporosis, inflammatory osteolysis, and other disorders characterized by excessive bone destruction.

## Data Availability

The original contributions presented in this study are included in the article/Supplementary Materials. Further inquiries can be directed to the corresponding author.

## Supplementary Materials

The following supporting information can be downloaded at: https://www.mdpi.com/article/10.3390/biomedicines14081716/s1, Table S1: Current evidence supporting the role of NLRP12 in osteoimmunology.

## Abbreviations

AMPK	AMP-activated protein kinase
AP	Apical periodontitis
ATP	Adenosine triphosphate
BMD	Bone mineral density
CTX	C-terminal telopeptide of type I collagen
DAMPs	Damage-associated molecular patterns
DCs	Dendritic cells
ERK	Extracellular signal-regulated kinase
FCAS2	Familial cold autoinflammatory syndrome type 2
HIF-1α	Hypoxia-inducible factor-1 alpha
IL	Interleukin
JNK	c-Jun N-terminal kinase
LPS	Lipopolysaccharide
MAPK	Mitogen-activated protein kinase
mTOR	Mammalian target of rapamycin
MyD88	Myeloid differentiation primary response protein 88
NACHT	NAIP, CIITA, HET-E, and TP1 domain
NEMO	NF-κB essential modulator
NFATc1	Nuclear factor of activated T cells 1
NF-κB	Nuclear factor kappa B
NLR	NOD-like receptor
NLRP3	NLR family pyrin domain containing 3
NLRP12	NLR family pyrin domain containing 12
NLRP12-AID	NLRP12-associated autoinflammatory disease
NOD	Nucleotide-binding oligomerization domain
OPG	Osteoprotegerin
P1NP	Procollagen type I N-terminal propeptide
PAMPs	Pathogen-associated molecular patterns
PANoptosis	Pyroptosis, apoptosis, and necroptosis (integrated inflammatory programmed cell death)
PBMCs	Peripheral blood mononuclear cells
PD	Periodontitis
PRRs	Pattern-recognition receptors
PYD	Pyrin domain
RA	Rheumatoid arthritis
RANK	Receptor activator of nuclear factor-κB
RANKL	Receptor activator of nuclear factor-κB ligand
ROS	Reactive oxygen species
scRNA-seq	Single-cell RNA sequencing
SNPs	Single-nucleotide polymorphisms
TAB2	TGF-β-activated kinase 1-binding protein 2
TAB3	TGF-β-activated kinase 1-binding protein 3
TAK1	Transforming growth factor-β-activated kinase 1
TGF-β	Transforming growth factor beta
Th17	T helper 17 cells
TLR	Toll-like receptor
TNF-α	Tumor necrosis factor alpha
TRAF3	TNF receptor-associated factor 3
TRAF6	TNF receptor-associated factor 6
Treg	Regulatory T cell
