# Supplementary material for: The NLRP12 Osteoimmune Checkpoint: Coordinating Inflammatory Signaling and Bone Remodeling
Source: Biomedicines. 2026 Jul 30;14(8):1716. doi: 10.3390/biomedicines14081716 (PMC13509648; doi:10.3390/biomedicines14081716)
Supplement: Supplementary file 1 [file biomedicines-14-01716-s001.zip › biomedicines-4427395-supplementary.pdf]

Supplemental Table S1. Current evidence supporting the role of NLRP12 in osteoimmunology

Category	Experimental Evidence	Human Evidence	Overall Strength	Major Knowledge Gaps
Osteoclasts	Extensive	Limited clinical evidence	Strong	Molecular regulation
Macrophages	Extensive	Moderate clinical evidence	Strong	Cell-specific mechanisms
Dendritic cells	Moderate	Limited clinical evidence	Moderate	Bone-specific functions
Neutrophils	Moderate	Limited clinical evidence	Moderate	Osteolysis
Osteoblasts	Limited	None reported	Weak	Expression and function
Osteocytes	Limited	None reported	Weak	Mechanotransduction
Treg/Th17 axis	Preliminary	None reported	Emerging	Adaptive immune regulation
Therapeutic targeting	Preclinical	None reported	Emerging	Drug development
